# Oriented mesoporous nanofibers show nature's wisdom

**DOI:** 10.1093/nsr/nwae152

**Published:** 2024-04-23

**Authors:** Yonghui Deng

**Affiliations:** Department of Chemistry, State Key Laboratory of Molecular Engineering of Polymers, Shanghai Key Laboratory of Molecular Catalysis and Innovative Materials, Collaborative Innovation Center of Chemistry for Energy Material (iChEM), Fudan University, China; State Key Lab of Transducer Technology, Shanghai Institute of Microsystem and Information Technology, Chinese Academy of Sciences, China

Electrodes assembled with conventional nanoparticulate materials in electrochemical energy devices usually have a random and torturous porous system. To meet ever-increasing demands for higher volumetric energy density at package level, packing more active materials in a limited volume becomes increasingly difficult since transportation of reactants (e.g. electrolyte) and charges through high-tortuosity channels/pathways in thick electrodes meets large impedance [1]. In nature, vascular bundles permit high-speed fluid dynamics of nutrients over long ranges [2]. Building vascular bundle-like structures of aligned channels with electron conduction highway over the whole electrode may resolve the issues of mass diffusion limitations. However, nanoscale biomimicry of vascular bundles has rarely been realized in energy devices due to the lack of a suitable synthetic methodology.

Taking inspiration from the vascular bundles in bamboo, a team led by Prof. Wei Luo at Donghua University has recently published an interesting work on preparing ordered mesoporous nanofibers (NFs) with well-aligned longitudinal mesopore channels through an electrospun bottom-up micelle self-assembly strategy [3]. Poly(ethylene oxide)-b–polystyrene (PEO-b-PS) block copolymer is prepared as a template and adjusted to form uniform PEO-b-PS/precursor monomicelles by adding polystyrene (PS) homopolymer as a pore expander. In contrast to the conventional evaporation-induced self-assembly (EISA) method, electrospinning produces a high-speed extensional flow of the micelle solution, in which strong hydrodynamic forces and fast evaporation of solvent are applied to achieve longitudinal stretching and close packing of micelles. This gives rise to continuous polymer nanofibers consisting of well-ordered axially-stretched micelles (Fig. 1). After removing the template, well-aligned parallel mesopores over centimeter scale can be formed in NFs and, more importantly, the pore size can be readily tuned from 12–19 nm *via* varying the PS content. Of note, the aligned mesopore channels are interconnected through tiny micropores, which is important for mass transport and active sites exposure. More interestingly, this approach allows large-scale fabrication of free-standing membranes, and can be generalized to fabricate ordered mesoporous NFs with various compositions such as carbon, SiO_2_, TiO_2_, and WO_3_. Luo's work demonstrates for the first time the ability of dynamic stretching to adjust the micelle structure, representing a great breakthrough for the synthetic methodology of mesoporous materials. By contrast, the conventional EISA method generates mesoporous materials mostly in powder form, and lack long-range order over the electrode scale.

**Figure 1.**
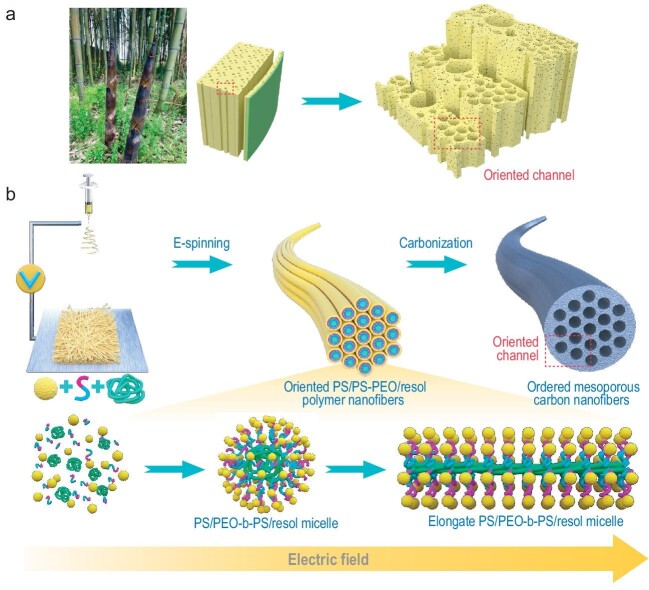
Schematics of bamboo's microscopic interior structure and electrospinning strategy of synthesizing oriented mesoporous nanofibers.

Using the structure–function integration of vascular bundles within the mesoporous carbon NFs, the team skillfully fabricated the NF-hosted lithium metal cathode, which is structurally stable, and enables uniform lithium nucleation and expedites Li^+^ transport. The Li ion diffusion coefficient of OD-MCNF (N, S) is about 20 times higher than the disordered counterpart. The ordered structure in OD-MCNF (N, S) could prolong Sand's time to ∼2600 minutes, which is about 1.5 times that of the disordered one. Such impressive improvements highlight the importance of achieving long-range order of mesoporosity in battery design. The corresponding lithium symmetric cell exhibits excellent cycling stability over 3000 h (3000 cycles) and ultra-low overpotentials of ∼15, ∼21 and ∼28 mV even at high current densities of 10, 20 and 30 mAh cm^−2^, outperforming recently reported cells. The assembled Li metal pouch cell delivers a cell-level mass energy density of 323 Wh kg^−1^.

In summary, this timely research article by Luo *et al*. reveals well-designed mesoporous NFs with oriented mesopores, demonstrating the power of nanoscale biomimicry of vascular structures for advanced electrochemical energy devices and beyond.

## References

[1] Janek J, Zeier WG. Nat Energy 2023; 8: 230–40. 10.1038/s41560-023-01208-9

[2] Tang T, Sui Z, Fei B. IAWA J 2022; 43: 322–36. 10.1163/22941932-bja10083

[3] Zhu X, Liu M, Bu F et al. Natl Sci Rev 2024; 11: nwae081. 10.1093/nsr/nwae081 38577675 PMC10989666

